# Acclimations to Cold and Warm Conditions Differently Affect the Energy Metabolism of Diapausing Larvae of the European Corn Borer *Ostrinia nubilalis* (Hbn.)

**DOI:** 10.3389/fphys.2021.768593

**Published:** 2021-11-22

**Authors:** Željko D. Popović, Vítězslav Maier, Miloš Avramov, Iva Uzelac, Snežana Gošić-Dondo, Duško Blagojević, Vladimír Koštál

**Affiliations:** ^1^Department of Biology and Ecology, Faculty of Sciences, University of Novi Sad, Novi Sad, Serbia; ^2^Department of Analytical Chemistry, Faculty of Science, Palacký University, Olomouc, Czechia; ^3^Maize Research Institute “Zemun Polje”, Belgrade, Serbia; ^4^Institute for Biological Research “Siniša Stanković”, Belgrade, Serbia; ^5^Biology Centre, Institute of Entomology, Academy of Sciences of the Czech Republic, České Budějovice, Czechia

**Keywords:** diapause, energy metabolism, ATP, NAD^+^, NADP^+^, COX, gene expression

## Abstract

The European corn borer *Ostrinia nubilalis* is a pest species, whose fifth instar larvae gradually develop cold hardiness during diapause. The physiological changes underlying diapause progression and cold hardiness development are still insufficiently understood in insects. Here, we follow a complex of changes related to energy metabolism during cold acclimation (5°C) of diapausing larvae and compare this to warm-acclimated (22°C) and non-diapause controls. Capillary electrophoresis of nucleotides and coenzymes has shown that in gradually cold-acclimated groups concentrations of ATP/ADP and, consequently, energy charge slowly decrease during diapause, while the concentration of AMP increases, especially in the first months of diapause. Also, the activity of cytochrome *c* oxidase (COX), as well as the concentrations of NAD^+^ and GMP, decline in cold-acclimated groups, until the latter part of diapause, when they recover. Relative expression of NADH dehydrogenase (*nd1*), coenzyme Q-cytochrome *c* reductase (*uqcr*), COX, ATP synthase (*atp*), ADP/ATP translocase (*ant*), and prohibitin 2 (*phb2*) is supressed in cold-acclimated larvae during the first months of diapause and gradually increases toward the termination of diapause. Contrary to this, NADP^+^ and UMP levels significantly increased in the first few months of diapause, after gradual cold acclimation, which is in connection with the biosynthesis of cryoprotective molecules, as well as regeneration of small antioxidants. Our findings evidence the existence of a cold-induced energy-saving program that facilitates long-term maintenance of larval diapause, as well as gradual development of cold hardiness. In contrast, warm acclimation induced faster depletion of ATP, ADP, UMP, NAD^+^, and NADP^+^, as well as higher activity of COX and generally higher expression of all energy-related genes in comparison to cold-acclimated larvae. Moreover, such unusually high metabolic activity, driven by high temperatures, lead to premature mortality in the warm-acclimated group after 2 months of diapause. Thus, our findings strongly support the importance of low temperature exposure in early diapause for gradual cold hardiness acquisition, successful maintenance of the resting state and return to active development. Moreover, they demonstrate potentially adverse effects of global climate changes and subsequent increase in winter temperatures on cold-adapted terrestrial organisms in temperate and subpolar regions.

## Introduction

Many organisms living in temperate and polar zones have evolved the ability to temporarily put their development on hold and survive harsh winter conditions by entering a specific form of resting state – diapause. A better understanding of the molecular processes behind diapause would be of high value, as it could help prolong the shelf-life of beneficial insects, lead to better pest management methods, improve cryopreservation techniques, offer answers to fundamental questions such as aging, obesity, transmission of diseases, and even become a rich source for the discovery and development of new pharmaceutical agents (Denlinger, 2008). Rather than being a static state of arrested ontogeny, diapause consists of successive phases with distinct gene expression patterns and physiological meanings – induction, preparation, initiation, maintenance, termination, and post-diapause quiescence (Koštál, 2006; Koštál et al., 2017). Diapausing insects arrest development and spend many months in inactivity exposed to various environmental stressors such as lack of food, thermal stresses (extreme cold spells, fluctuations of temperature), risk of freezing of body fluids, loss of body water, and exposure to pathogens and predators (Storey and Storey, 2004, 2007, 2012; Blagojević, 2007; Staples and Buck, 2009; MacRae, 2010). In addition to external factors, the diapausing insects are challenged by various internal problems such as impaired production of energy, reduced activity of membrane ion pumps resulting in dissipation of transmembrane ion potentials, and increased production of reactive oxygen and nitrogen species (ROS/RNS). For all these reasons, deep molecular, biochemical, and physiological alterations need to occur during diapause in order to support enhanced stress tolerance (MacRae, 2010). During diapause progression, major metabolic alterations occur, including: amassing of energy reserves (Hahn and Denlinger, 2007) followed by depression of metabolism in order to preserve energy (Harvey, 1962; Hahn and Denlinger, 2011), lipid rearrangements (Vukašinović et al., 2013), and transcription of specific gene sets (Denlinger, 2002; Robich et al., 2007; MacRae, 2010; Koštál et al., 2017) during the prolonged phase of arrested development.

The European corn borer *Ostrinia nubilalis* (Hübner, 1796), originally a Eurasian species, was accidentally introduced to North America and North Africa in the early 20th century (Krumm et al., 2008). Its larvae are known to feed on more than 200 plant species, most notably grains, vegetables and fruits which are economically important (Molinari et al., 2010; Robinson et al., 2010). The fifth (final) instar larvae enter diapause which is photoperiodically induced in response to shortening day length during September. Diapausing larvae will overwinter until they return to active development in April (Beck, 1962). As a response to decreasing ambient temperatures, the fat body of diapausing larvae begins to synthesize glycerol which is exported and accumulated in the hemolymph. This leads to the lowering of the insect’s supercooling point to below −20°C and the development of cold hardiness (Nordin et al., 1984; Grubor-Lajšić et al., 1991; Andreadis et al., 2008). Despite the fact that diapause and cold hardiness in *O. nubilalis* have been extensively studied at physiological and biochemical levels (Beck et al., 1969; Beck and Shane, 1969; Beck, 1989; Grubor-Lajšić et al., 1991; Stanić et al., 2004; Jovanović-Galović et al., 2007; Kojić et al., 2009, 2018; Blagojević et al., 2011; Vukašinović et al., 2013, 2015, 2018; Purać et al., 2015; Uzelac et al., 2020), the exact molecular processes driving diapause development and increased stress-tolerance in this species remain insufficiently understood.

Owing to the ongoing climate changes, we have witnessed the rise of global temperatures, even during the winter season in temperate, subpolar, and polar regions. In light of that, the main goal of this study was to investigate how warm acclimation could reflect on the energy metabolism of diapausing larvae of *O. nubilalis*, a species that is naturally adapted to cold winter temperatures in its habitats.

## Materials and Methods

### Insect Rearing and Experimental Design

**FIGURE 1:**
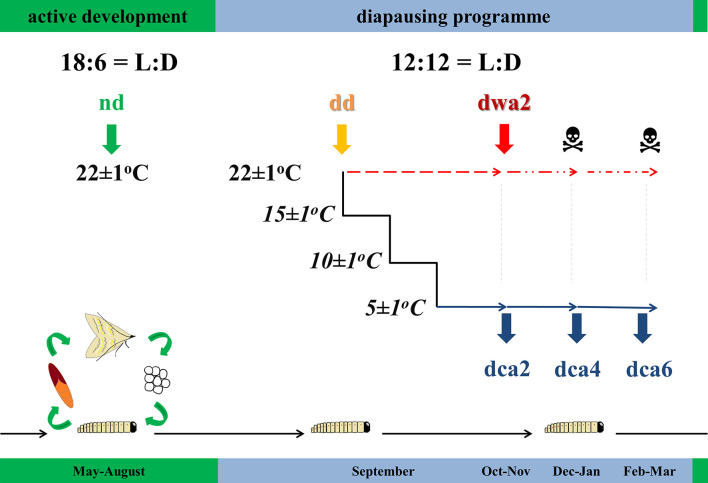
Schematic illustration of experiment (explanation given in the text): *nd* – non-diapause; *dd* – diapause-destined; *dwa2* – 2 months diapause, warm-acclimated (22°C); *dca2* – 2 months diapause, *dca4* – 4 months diapause, *dca6* – 6 months diapause, cold-acclimated (5°C); L – light, D – dark. The timeline given below the experimental design represents the typical life cycle of *O. nubilalis* in field conditions in temperate climate.

European corn borer eggs were obtained from the Maize Research Institute “Zemun Polje” in Belgrade, Serbia. Egg patches were sterilized with 4% Persteril solution (Overlack, Czech Republic), placed in translucent polycarbonate boxes with a semi-artificial diet (Salama, 1970) and were reared under different light:dark (LD) regimes at 22 ± 1°C. Non-diapausing larvae were reared under the photoperiod of 18L:6D, while diapause was induced with the photoperiod of 12L:12D. Larvae were observed on a daily basis and reared under the specified conditions until they reached the wandering stage of the final instar, when non-diapause (*nd*) and diapause-destined (*dd*) larvae were sampled (Figure 1).

Furthermore, diapause-destined larvae were either allowed to enter diapause at 22 ± 1°C (warm-acclimated) or were gradually chilled to 5 ± 1°C (−2°C/day) and then allowed to become dormant (cold-acclimated). Diapausing larvae were sampled every 2 months (Figure 1), except for warm-acclimated larvae that were sampled only once due to the high mortality (>95%) after 3 months in diapause. Overall, six experimental groups were established (Figure 1): non-diapause (*nd*), diapause-destined (*dd*); warm-acclimated, 2 months diapause (*dwa2*); cold-acclimated, 2 (*dca2*), 4 (*dca4*), and 6 (*dca6*) months diapause.

### Capillary Electrophoresis

All chemicals were of analytical-reagent grade. Citric acid, γ-aminobutyric acid (GABA), cetyltrimethylammonium bromide (CTAB) and standards of nucleotides were obtained from Sigma (St. Louis, United States), methanol from Fluka (Buchs, Switzerland), and deionised water (18.2 Ωcm) from Elga Bucks, United Kingdom. Running electrolyte was prepared by dissolving citric acid to a concentration of 40 mM in water and titrating to pH 4.4 with solid GABA. Cationic surfactant CTAB was then added to a final concentration of 8 mM CTAB, and the solution was sonicated for 15 min at ambient temperature to thoroughly mix the surfactant with the running electrolyte. The standard solutions of each nucleotide were prepared separately by dissolving each nucleotide in deionized water (5 mg/mL). The standard mixture of nucleotides in the concentration of 1.0 mg/mL was prepared by mixing and diluting PAD standards with deionized water. Lower concentrations were prepared by diluting the mixture in deionized water.

#### Sample Preparation

Three larvae per biological pool were homogenized on ice in pre-chilled 50 mM phosphate buffer pH 7 with a Teflon/glass homogenizer at 1,500 rpm with 10 strokes. For deproteination, 100 μL of crude 30% homogenate was transferred into a clean vial containing 300 μL methanol, vigorously vortexed for 30 s and sonicated for 3 min. The alcohol-sample mixture was centrifuged at 10,000 rpm for 5 min and the supernatant was transferred into a clean vial and evaporated under nitrogen stream. The residue was reconstituted with 100 μL of deionised water (18.2 Ωcm, Elga Bucks, United Kingdom), directly injected into the capillary and analyzed for the presence of various nucleotides and coenzymes. Separations were performed on the capillary electrophoresis (CE) system HP 3DCE (Agilent Technologies, Waldbronn, Germany) as described in Friedecký et al. (2007). Nucleotide concentrations were expressed as mol/mg of fresh mass. Energy charge (EC) of adenine nucleotides, which reflect the rate of metabolism, was calculated using the standard equation (Atkinson, 1968):


$$\text{EC}=\frac{\left[\text{ATP}\right]+\frac{1}{2}\,\left[\text{ADP}\right]}{\left[\text{ATP}\right]+\left[\text{ADP}\right]+\left[\text{AMP}\right]}$$

#### Capillary Electrophoresis Apparatus and Running Conditions

Capillary electrophoresis system HP 3D Agilent (Waldbronn, Germany) equipped with on-column diode array detector was used. The separation was performed in a fused silica capillary of 80.5 cm total length, 72 cm effective length and 75 μm width (MicroSolv Technology Corporation, NJ, United States). The capillary was set to 25°C and rinsed for 15 min with 0.1M NaOH, then for 15 min with deionized water and then with the running electrolyte at the beginning of each work day. Between individual analyses the capillary was washed for 1 min with 0.1M NaOH, then for 1 min with deionized water and then for 2 min with the running electrolyte. The detection wavelength was set to 260 nm. Injection was performed under 50 mbar/20 s of pressure and −25 kV of voltage. All measurements were performed five times unless stated otherwise.

### Activity of Cytochrome *c* Oxidase

Cytochrome *c* oxidase (COX) activity was assessed by monitoring the decrease in absorbance at 550 nm as described in Hofmann and Hand (1990). Reduced cytochrome *c* from the equine heart (RHCC) was prepared as described by Eddy et al. (2006) and stored at −20°C for up to 1 month. Before beginning the measurements, the stability of the reagent was validated by scanning the 450–600 nm spectrum. Ten frozen larvae per biological pool were homogenized on ice in 50 mM phosphate buffer pH 7 crushed (20% w:v) with a Teflon/glass homogenizer at 1,500 rpm with 10 strokes. The crude homogenate was transferred into a new tube, incubated on ice for 10 min and centrifuged at 1,000 *g* for 10 min to remove cellular debris. Supernatants were removed, placed into new tubes and centrifuged at 20,000 *g* for 20 min to pellet mitochondria. The pellet was washed with phosphate buffer, used for homogenization, and centrifuged again at 20,000 *g* for 20 min for further purification. The mitochondria precipitate was dissolved in the same phosphate buffer with added 0.5M sucrose, frozen in liquid nitrogen and stored at −80°C until analysis. Protein concentration was determined by Quick Start BioRad Assay (BioRad, Hercules, CA, United States) and bovine serum albumin was used as the standard. COX activity was measured at 550 nm using the Infinite M200 UV/VIS spectrophotometer/fluorimeter (Tecan Group ltd). One 250 μL reaction mixture contained phosphate buffer and 50 μM of RHCC. Reactions were initiated by adding up to 10 μL of mitochondrial fraction and the decrease in absorbance was recorded. Blanks omitted RHCC and conditions were optimized to ensure a linear reaction rate. One unit of enzyme activity was defined as the amount of enzyme that oxidized 1 nmol of RHCC per minute. All measurements were performed in technical duplicates for every biological pool. Data are expressed as units of enzyme activity per mg of total protein.

### Quantitative Real-Time PCR

#### RNA Extraction and cDNA Synthesis

Fat body tissue was dissected and pooled from five larvae per biological replicate and total RNA was extracted using the RNA Blue kit (TopBio, Prague, Czech Republic), according to the manufacturer’s instructions. Concentrations of total RNA were measured using NanoDrop 2000 (ThermoScientific, Waltham, MA, United States), while the quality was assessed on 2% agarose gel electrophoresis. Concentrations of total RNA were adjusted to 1 μg/μL in all samples and overall 5 μg of total RNA were used for the first strand synthesis with the Reverse Transcription System (Promega, Madison, WI, United States). Obtained cDNA was diluted 25 times with ultrapure water and used in the measurement of relative abundance of mRNA transcripts by quantitative real-time PCR (qRT-PCR). PCR reactions were primed with a pair of gene-specific oligonucleotides (Table 1) that were generated using Primer3 software (Untergasser et al., 2012).

**TABLE 1:** Primer pairs used for relative gene expression analysis, with their accession numbers and primer sequences.

**Gene coding for**	**GeneBank accession no.**	**Primer (F – forward, R – reverse)**	**5′–3′ primer sequence**
Actin (reference #1)	EL928709.1	On_actin_F	CAGAAGGAAATCACAGCTCTAGCC
		On_actin_R	ATCGTACTCCTGTTTCGAGATCCA
Ribosomal protein S03 (reference #2)	EL929086.1	On_rps3_F	GTCGCAGAATTCGTGAACTAACCT
		On_rps3_R	ATGATGAACCTCAGCACACCATAG
NADH dehydrogenase 1 (complex I)	AF349037.1	On_Nadh1_F	CGGGCAGTAGCTCAAACTATTTCAT
		On_Nadh1_R	GAAACTAATTCTCTCTCACCTTCAGCA
Coenzyme Q – cytochrome *c* reductase (complex III)	EL928629.1	On_ubiq_CytC_F	GTCTCTTGCGTCTTCAGGATTAACA
		On_ubiq_CytC_R	CTCTTCGAATATGGCTGTGGTATTG
Cytochrome *c* oxidase (complex IV)	EU128659.1	On_CO1_F	TTAGGGCTAGCTGGTATACCTCGAC
		On_CO1_R	TGAATGTTCTGCTGGTGGTAGATTT
ATP synthase γ (complex V)	EL929482.1	On_ATPsyn_F	GTTAACAACCAGCAGAACCGTAACA
		On_ATPsyn_R	ATACAGTTGCTTGGGCTCATCTTCT
ADP/ATP translocase	EL928491.1	On_ANT_F	GAAGACCAGCGCTACAAAGGTATC
		On_ANT_R	CAGAACTGCGTCTTCTTGTCTACG
Prohibitin 2	EL930118.1	On_Phb2_F	ACAGGTCTCCTTGCTGATTAGACG
		On_Phb2_R	GCTTTGCTCTTTCCACAACAAAAG

#### Quantitative Real-Time PCR and Relative Gene Expression

IQ SYBR Green Supermix was used to prepare the real-time PCR reactions, according to the manufacturer’s instructions (BioRad, Hercules, CA, United States). Threshold cycles (Ct) and melting curves were recorded using the CFX96 Real-Time PCR System (BioRad, Hercules, CA, United States). Each experimental group consisted of five biological replicates and each sample was run in technical duplicates. Relative gene expression was calculated according to the method by Ganger et al. (2017) and expressed in ΔCt values using the following equation:


$$\Delta \,\text{Ct}=\left(\left(\left(\log_{10}\,{\text{E}}_{\mathrm{ref1}}*{\text{Ct}}_{\mathrm{ref1}}\right)+\left({\text{log}}_{10}\,{\text{E}}_{\mathrm{ref2}}*{\text{Ct}}_{\mathrm{ref2}}\right)\right)/2\right)-\left(\log_{10}{\text{E}}_{\text{Goi}}*{\text{Ct}}_{\text{Goi}}\right)$$

where *E* is primer efficiency, *ref1* and *ref2* are the reference genes (*rps3* and *actin*, respectively) and *Goi* is the gene of interest. The calculated ΔCt values were used for statistical analysis and shown as univariate scatterplots, as is recommended for small sample size studies (Weissgerber et al., 2015).

### Statistical Tests

All analyses were performed in technical duplicates for each biological pool. Statistical analysis was performed on the means of the technical duplicates using the data analysis software system Statistica version 13 (StatSoft, Inc., Tulsa, OK, United States). The statistical significance of the differences between the larval experimental groups was tested using one-way ANOVA followed by *post hoc* Tukey’s test, with a level of significance of *p* < 0.05. Additionally, principal component analysis (PCA) was used to analyse the data.

## Results

### Capillary Electrophoresis of Nucleotides and Coenzymes

**FIGURE 2:**
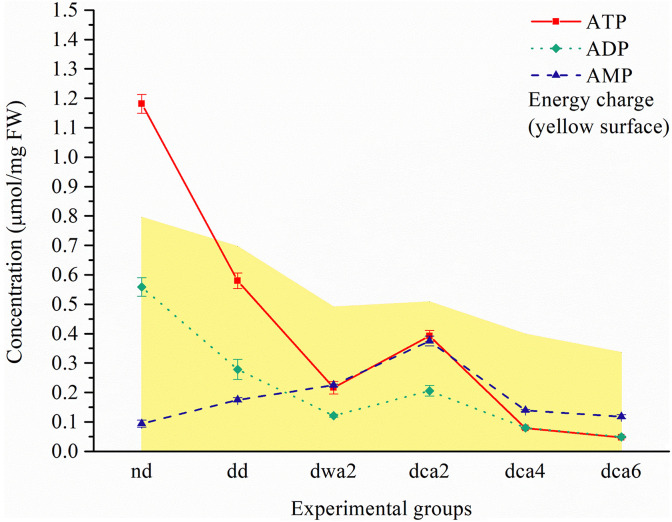
Adenine nucleotide concentration and energy charge (yellow surface) in *O. nubilalis* larvae. Results are expressed as mean concentration ± standard error obtained for 3 independent pools (5 larvae per pool). The lines connecting the data points were added to improve visual clarity and help picture the general trend of the results.

**FIGURE 3:**
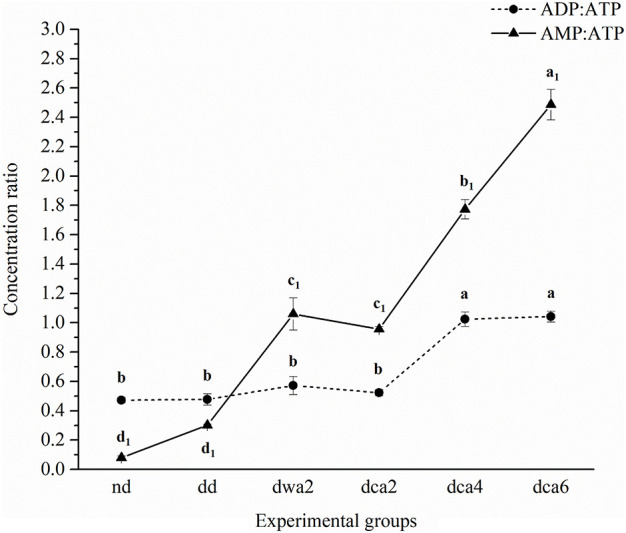
Concentration ratios of different adenine nucleotides in *O. nubilalis* larvae. Results are expressed as mean concentration ± standard error obtained for 3 independent pools (5 larvae per pool). The statistical significance of determined values was tested using one-way ANOVA followed by *post hoc* Tukey’s test, with a level of significance of *p* < 0.05. Statistically significant results are labeled with different letters above the data points. Non-indexed letters refer to ADP:ATP results and indexed letters refer to AMP:ATP results. The lines connecting the data points were added to improve visual clarity and help picture the general trend of the results.

**FIGURE 4:**
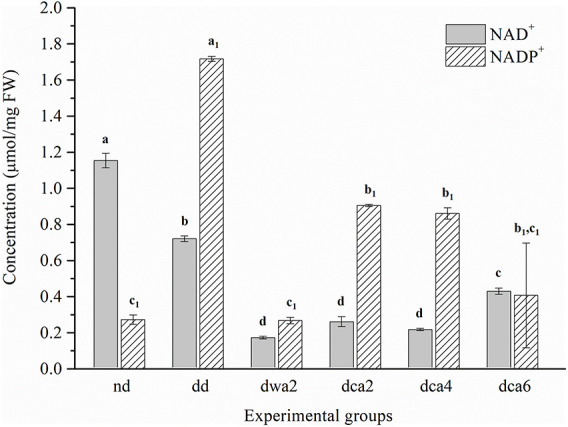
Nicotinamide adenine dinucleotide (NAD^+^, NADP^+^) concentration in *O. nubilalis* larvae. Results are expressed as mean concentration ± standard error obtained for 3 independent pools (5 caterpillars per pool). The statistical significance of determined values was tested using one-way ANOVA followed by *post hoc* Tukey’s test, with a level of significance of *p* < 0.05. Statistically significant results are labeled with different letters above the bars. Non-indexed letters refer to NAD^+^ results and indexed letters refer to NADP^+^ results.

Five distinct nucleotides (ATP, ADP, AMP, UMP, and GMP) and two coenzymes (NAD^+^ and NADP^+^) were detected and quantified in all experimental groups using CE (Figures 2–4). Moreover, the EC and ratios of the three distinct adenosine-phosphate nucleotides were determined.

#### Adenine Nucleotides and Energy Charge

The results of CE showed that the concentrations of ATP and ADP were high in non-diapausing and low in diapause-destined and diapausing larvae (Figure 2). In cold-acclimated diapausing larvae, the concentrations of adenine nucleotides were the highest at the beginning of diapause (*dca2*), after which they gradually decreased. The EC was the highest in non-diapausing larvae (∼0.8), decreasing in diapausing groups and reaching the lowest levels at the end of diapause (∼0.4). The concentration of AMP was the lowest in non-diapausing larvae, and the highest in the *dca2* group, after which it gradually decreased. In warm-acclimated diapausing larvae (*dwa2*), the amount of ATP and ADP nucleotides was lower in comparison to the cold-acclimated group in the same period of diapause (*dca2*; Figure 2).

Furthermore, ADP:ATP and AMP:ATP ratios were calculated (Figure 3). It was found that ADP:ATP and AMP:ATP ratios were substantially higher in diapausing groups in comparison to non-diapausing. Also, both ADP:ATP and AMP:ATP ratios increased significantly during the time course of diapause. The increase of AMP:ATP ratio was more pronounced even in the diapause-destined group compared to the non-diapause group.

#### NAD^+^ and NADP^+^

The concentrations of NAD^+^ and NADP^+^ coenzymes differ substantially between the experimental groups (Figure 4). The concentration of the NAD^+^ coenzyme was higher in non-diapausing and diapause-destined larvae, and lower in diapausing larvae. At the end of diapause (*dca6*), the amount of NAD^+^ doubled compared to the beginning of diapause (*dca2*). On the other hand, the concentration of the NADP^+^ coenzyme was low in non-diapausing, the highest in diapause-destined and had a decreasing trend in diapausing larvae. It was found that the concentration of NADP^+^ was higher in the first months of the diapause (*dca2*, *dca4*), and at the end of the diapause it decreased (*dca6*). The concentrations of both analyzed coenzymes were lower in diapausing larvae acclimated to higher temperatures (*dwa2*) compared to cold-acclimated diapausing larvae (*dca2*).

**FIGURE 5:**
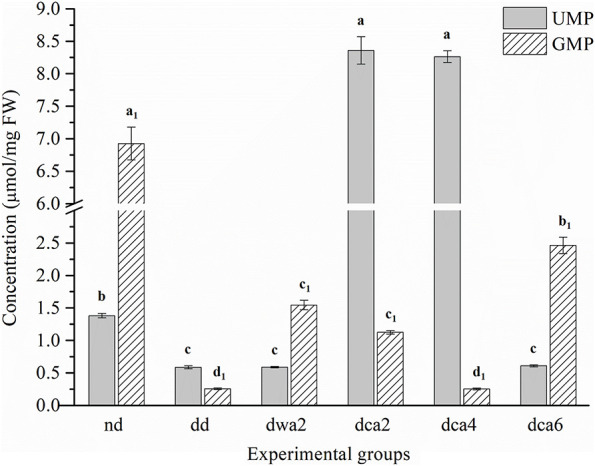
The concentration of UMP and GMP nucleotides in *O. nubilalis* larvae. Results are expressed as mean concentration ± standard error obtained for 3 independent pools (5 larvae per pool). The statistical significance of determined values was tested using one-way ANOVA followed by *post hoc* Tukey’s test, with a level of significance of *p* < 0.05. Statistically significant results are labeled with different letters above the bars. Non-indexed letters refer to UMP results and indexed letters refer to GMP results.

Measured concentrations of UMP and GMP nucleotides were significantly different between the experimental groups (Figure 5). The concentration of UMP was relatively low in all groups except in the diapausing groups *dca2* and *dca4*, where the concentration was increased several times. At the end of diapause, UMP concentration decreased significantly (*dca6*). In contrast, the concentration of GMP was the highest in non-diapausing larvae and lower in diapause-destined larvae. In diapause, the concentration of GMP was moderately high after 2 months in diapause (*dwa2*, *dca2*), decreased after 4 months (*dca4*) and rose significantly at the end of diapause (*dca6*).

### Cytochrome *c* Oxidase

**FIGURE 6:**
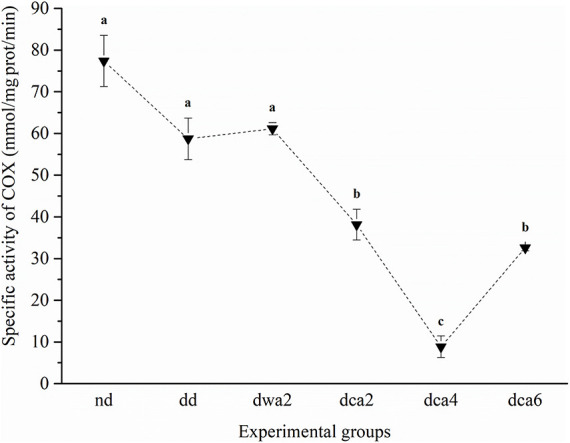
Specific activity of COX in *O. nubilalis* larvae. Results are expressed as mean ± standard error obtained for 3 independent pools (5 larvae per pool). The statistical significance of determined values was tested using one-way ANOVA followed by *post hoc* Tukey’s test, with a level of significance of *p* < 0.05. Statistically significant results are labeled with different letters above the data points. The dashed line connecting the data points was added to improve visual clarity and help picture the general trend of the results.

The specific activity of COX significantly differed between experimental groups (Figure 6). The activity of COX was higher in larvae reared and acclimated to 22°C (*nd*, *dd*, *dwa2*) in comparison to groups acclimated to 5°C (*dca2*, *dca4*, *dca6*). It was the highest in non-diapausing larvae and the lowest in the middle of diapause (*dca4*), after which it increased in the latter part of diapause. COX activity differed significantly among diapausing larvae that have been acclimated under two distinct temperature regimes for 2 months (*dwa2* and *dca2*) – it was higher in the group reared at 22°C (*dwa2*).

### Expression of Energy-Related Genes

**FIGURE 7:**
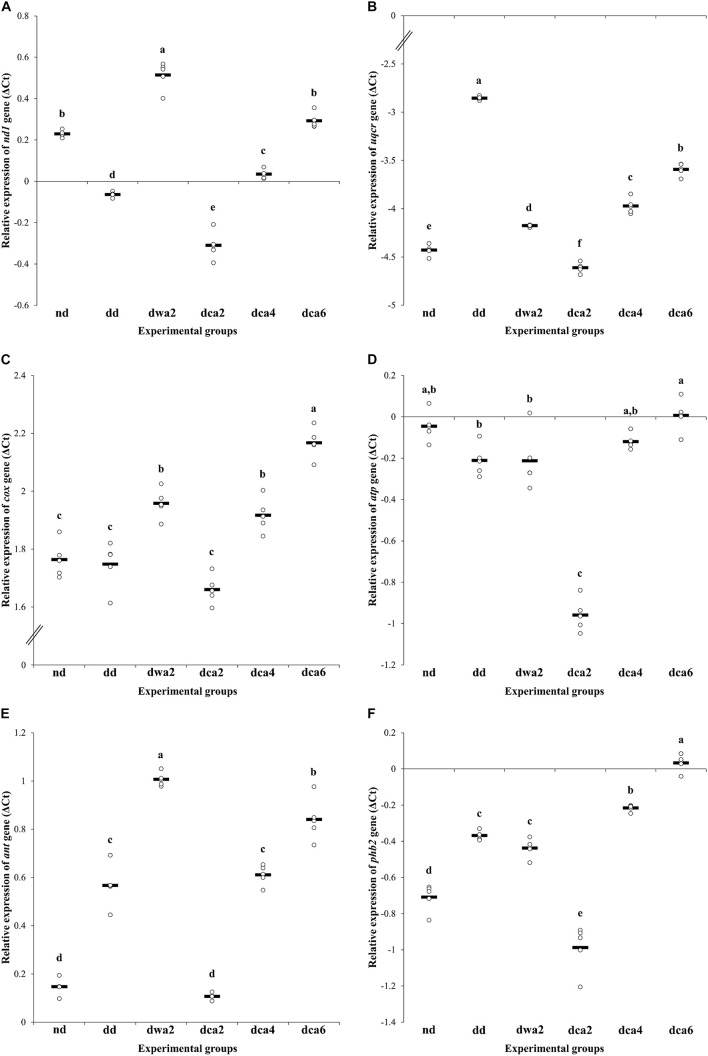
Relative gene expression of: NADH dehydrogenase subunit 1 **(A)**, CoQ-cytochrome *c* reductase subunit 9 **(B)**, cytochrome *c* oxidase subunit 1 **(C)**, ATP synthase subunit γ **(D)**, ADP/ATP translocase **(E)**, and prohibitin 2 **(F)** in the fat body of *O. nubilalis* 5th instar larvae. Ct values obtained from qRT-PCR were normalized to ribosomal protein S03 and actin. All results are expressed in ΔCt and presented as univariate scatterplots, where each dot represents one biological pool comprised of fat bodies from 5 larvae. The statistical significance of determined values was tested using one-way ANOVA followed by *post hoc* Tukey’s test, with a level of significance of *p* < 0.05. Statistically significant results are labeled with different letters above the scatterplots.

The relative gene expression of individual subunits of the electron transport chain in the fat body of larvae differed both between the experimental groups and between the analyzed genes (Figure 7). When compared to the non-diapausing group, the expression of CoQ-cytochrome *c* reductase and COX genes was significantly increased in diapausing groups (Figures 7B,C). On the other hand, the expression of NADH dehydrogenase and ATP synthase genes was reduced at the beginning of diapause, and then gradually increased, reaching the same level at the end of diapause as in non-diapausing larvae (Figures 7A,D).

Gene expression in diapause-destined larvae was different and specific for each gene. Expression of COX and ATP synthase genes was similar to that in the non-diapausing group, while the expression of NADH dehydrogenase and CoQ-cytochrome *c* reductase genes was increased in comparison to *nd* larvae (Figures 7A–D).

In diapausing larvae, the relative expression of all analyzed genes in the electron transport chain had a characteristic pattern – it was higher in the warm-acclimated group (*dwa2*) when compared to the cold-acclimated group (*dca2*). Going further, it usually followed a trend of gradual increase in gene expression during the time course of diapause (Figures 7A–D).

When it comes to *ant* and *phb2*, these two genes share a similar pattern of expression, with statistically significant differences between experimental groups. Compared to non-diapausing larvae, both of these genes are up-regulated in diapausing groups, except in cold-acclimated larvae in early diapause (*dca2*). In this group, gene expression is either unchanged (*ant*) or lowered (*phb2*) in comparison to non-diapausing larvae (Figures 7E,F).

In diapausing larvae, both genes exhibit a similar expression pattern – lowest expression in the *dca2* group, with gradual increase until the highest expression is reached near the end of diapause (*dca6*). Also, as shown in Figures 7E,F, both of these genes have much higher expression in warm-acclimated larvae in early diapause (*dwa2*) compared to larvae of the same age, but cold-acclimated (*dca2*).

### Principal Component Analysis

**FIGURE 8:**
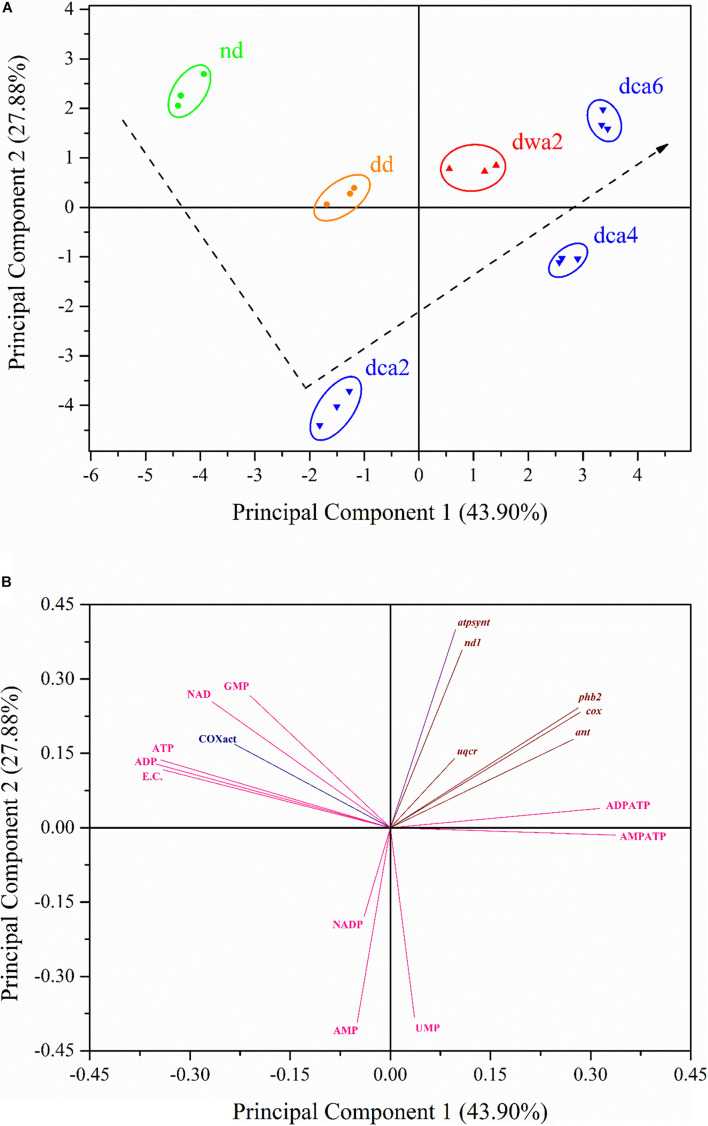
Plots of component scores (symbols; **A**) and of the variable loadings (vectors; **B**) for the two principal components from PCA performed on the activity of COX, concentrations of nucleotides and coenzymes, energy charge, adenine nucleotide ratios, as well as relative expression of analyzed genes. The dashed line illustrates the supposed trend of changes in metabolic rate between the experimental groups. *nd* – non-diapause; *dd* – diapause-destined; *dwa2* – 2 months diapause, warm-acclimated (22°C); *dca2* – 2 months diapause, *dca4* – 4 months diapause, *dca6* – 6 months diapause, cold-acclimated (5°C). Scores are scaled by the square root of the eigenvalues (i.e., scaling = 2).

In order to determine where the variation in the dataset comes from, as well as to reveal what links the patterns of the performed measurements together, the gathered data was subjected to PCA and the results are presented in Figure 8. The PCA score plot grouped the samples into clusters according to their similarity (Figure 8A), while the PCA loading plot (Figure 8B) shows how each principal component is affected by the analyzed data. As can be seen in Figure 8, the PCA extracted two major principal components which cover 71.78% of the data variance. The first principal component (PC1) accounted for 43.90% of the total variance and it separated the *nd*, *dd*, and *dca2* groups (negative scores) from the *dwa2*, *dca4*, and *dca6* groups (positive scores). Based on this, it is highly likely that PC1 accounts for the variance caused by the time course of diapause (Figure 8A). Furthermore, PC1 was positively correlated with the expression of all analyzed genes, as well as UMP concentration and adenine nucleotide ratios. Negative correlations were observed for COX enzyme activity, EC content and the remaining nucleotide concentrations (Figure 8B). The second principal component (PC2) accounted for 27.88% of the total variance. It is likely that this component reflects the effect of metabolism rates on the analyzed characteristics, considering that it separated the metabolically less active *dca2* and *dca4* groups (negative scores) from the rest (Figure 8A). PC2 showed positive correlation with most of the analyzed characteristics, except for the concentrations of UMP, AMP, and NADP^+^, as well as AMP:ATP ratios (Figure 8B).

## Discussion

Diapause is defined as a period of arrested development during which organisms cope with many stressful factors such as dehydration, high/low temperatures, lack of oxygen (Storey and Storey, 2007; MacRae, 2010). In larval diapause of *O. nubilalis* exposure to low temperatures increases the production of ROS, such as hydrogen peroxide (Kojić et al., 2009). Thus, the antioxidative defense system (ADS) in this species is connected with other biochemical adaptations to winter season conditions, such as the biosynthesis of polyol cryoprotectants (Grubor-Lajšić et al., 1997; Jovanović-Galović et al., 2004, 2007; Stanić et al., 2004; Popović et al., 2015; Uzelac et al., 2020). In addition, the ADS is supported by intensive pentose phosphate pathway (PPP) activity during the first months of diapause in *O. nubilalis*, since this pathway is a unique source of NADPH in the cell (Stanić et al., 2004). Also, the suppression of metabolism during this period of dormancy is well-known and has been recorded as a decrease in the activity of many mitochondrial enzymes, such as citrate synthase, NAD^+^-isocitrate dehydrogenase, glutamate dehydrogenase, COX (Joanisse and Storey, 1994). Similarly, in *O. nubilalis* larvae, oxygen consumption is up to four times lower during the winter diapause in comparison to the period of active development (Beck and Hanec, 1960). Here we presented different effects of cold and warm acclimation on the energy status of diapausing *O. nubilalis* larvae. The energy status was monitored by determining: the concentration of adenine nucleotides (ATP, ADP, and AMP), their ratios (EC), the activity of COX, as well as the concentration of NAD^+^, NADP^+^, UMP, and GMP, and relative expression of genes encoding proteins involved in mitochondrial biogenesis and energy metabolism.

### Cold-Acclimation Suppresses Metabolism and Enables Long-Term Maintenance of Diapause

ATP is a universal energy tender in living systems and cells must maintain a high ratio of ADP:ATP and AMP:ATP nucleotides in order to preserve homeostasis. The AMP:ATP nucleotide ratio is particularly important, as these adenine nucleotides are known to be major allosteric enzyme modulators, but most often with opposing effects (Hardie and Hawley, 2001). In comparison to non-diapause, ATP/ADP concentrations and EC decrease in diapause-destined and all diapausing groups, regardless of temperature acclimation. Conversely, AMP concentration increases in diapause, especially in the cold-acclimated group that was dormant for 2 months (*dca2*), as also supported by the PCA. Thus, the ADP:ATP and AMP:ATP ratios are higher in diapause groups, compared to non-diapause groups. Since low levels of ATP/ADP and high levels of AMP are characteristic of early diapause at both high (*dwa2*) and low temperatures of acclimation (*dca2*), these results indicate endogenous regulation of the consumption of energy equivalents during diapause. However, it should be noted that adenine nucleotide concentrations were two times higher in the cold-acclimated (*dca2*) group compared to the warm-acclimated group (*dwa2*). Similarly, the activity of COX, concentrations of NAD^+^ and GMP, as well as the expression of all analyzed energy metabolism-related genes decrease during the first months of diapause in cold-acclimated larvae. Our findings are consistent with high levels of AMP and lower levels of ATP/ADP obtained in similar studies on dormant phases of the killifish *Austrofundulus limnaeus* (Podrabsky and Hand, 1999) and in the embryos of the shrimp *Artemia franciscana* (Zhu et al., 2009). Given the scarcity of literature data on the participation of GMP in diapause, the obtained results could be explained by assuming possible functions of a physiologically important form of GMP nucleotide – cyclic GMP (cGMP), which unfortunately could not be quantified separately from the non-cyclical form by CE in this study. Thus, we can assume that in non-diapausing larvae cGMP probably serves as a secondary messenger in various processes of active metabolism, while in diapausing larvae it is less important due to hypometabolism, until the termination of diapause and the forthcoming metamorphosis. Namely, in the flesh fly *Sarcophaga crassipalpis*, diapause was shown to be interrupted when cGMP or its derivatives were injected into diapausing pupae (Fujiwara and Denlinger, 2007), and in the moth *Mamestra configurata*, cAMP was found to participate in maintaining diapause, while cGMP brought on its termination (Bodnaryk, 1987). In the roundworm *Caenorhabditis elegans*, cGMP has been found to regulate nutrition and, based on nutrient availability, affect dormancy, allowing the body to survive adverse conditions (You et al., 2008). The concentration of NAD^+^ was expected to be reduced in cold-acclimated diapausing larvae with suppressed metabolism, in comparison to actively developing larvae with intensive respiration and metabolism. As NADH/NAD^+^ are the main carriers of reduced equivalents for the electron transport chain in mitochondria, a decrease in NAD^+^ concentrations in diapause was expected. Since *O. nubilalis* larvae do not feed during diapause and subsequent exposure to low temperatures the number of mitochondria and oxygen consumption decrease, and so does their metabolic rate (McLeod and Beck, 1963; Jovanović-Galović, 1997). Cold induced metabolic suppression in diapause of *O. nubilalis* was also supported by lower COX activity in diapausing compared to non-diapausing groups. Similar findings were obtained in the cotton bollworm *Helicoverpa armigera* (Yang et al., 2010), the shrimp *A. franciscana* (Reynolds and Hand, 2004), the moth *Omphisa fuscidentalis* (Singtripop et al., 2007), as well as in the moth species *Epiblema scudderiana* and the fly *Eurosta solidaginis* (McMullen and Storey, 2008). Moreover, in *E. scudderiana* and *E. solidaginis*, COX activity was found to decrease further with exposure of diapausing individuals to low temperatures (McMullen and Storey, 2008).

As opposed to GMP and NAD^+^, UMP and NADP^+^ concentration increased significantly in the first months of diapause (*dca2*, *dca4*), and then decreased ahead of diapause termination. Interestingly, the increase in NADP^+^ was observed already in *dd* larvae, which may signal preparation for diapause and the forthcoming metabolic depression. The concentration of UMP nucleotides in these groups was increased 10-fold and returned to diapause-destined levels at the end of diapause. The high concentration of NADP^+^ coenzymes in the initial months of diapause is in agreement with previous studies of diapause in the European corn borer, which showed that there is a close connection between glycerol synthesis, hexose-monophosphate shunt activity and the antioxidant protection system (Stanić et al., 2004). Hexose-monophosphate shunt activity and NADPH synthesis were found to increase in the initial months of diapause. During diapause, NADPH serves as a source of reducing equivalents in antioxidant protection, representing a substrate for the regeneration of antioxidant molecules such as GSH, thioredoxin, glutaredoxin, and others. Also, NADPH is necessary for biosynthetic processes, such as the production of glycerol which participates in the protection of cells from low temperatures (Stanić et al., 2004; Blagojević, 2007; Kojić et al., 2010, 2018; Purać et al., 2015; Uzelac et al., 2020). In addition to the concentration increase of the NADP^+^ in *dca2* and *dca4* larvae, a significant increase in the transcription of the thioredoxin (*trx*) gene was found in the same groups (Popović et al., 2015).

On the other hand, the importance of UMP nucleotides in insect development and dormancy is poorly described in literature. However, elevated uridine plasma levels have been shown after fasting in mice/rat models and humans, and the levels dropped rapidly after the fast was broken (Deng et al., 2017). Also, in these mammalian models, ATP depletion leads to increased levels of UMP and UDP, since ATP is a phosphate donor for UTP synthesis (Zhang et al., 2020). For this reason, UMP can be viewed either as a precursor for UDP nucleotide synthesis or, conversely, as a product of UDP hydrolysis in the ADP phosphorylation reaction and ATP regeneration. UDP nucleotides, as components of UDP-glucose, participate in the synthesis of trehalose and glycogen (Murphy and Wyatt, 1964). In *O. nubilalis* larvae, glycogen synthesis is intensive before entering diapause, after which glycogen serves as a source of glucose that is necessary for energy production, as well as for glycerol synthesis during diapause (Grubor-Lajšić et al., 1991). In addition to glycerol, trehalose is intensively synthesized in the initial months of diapause, as it is an important component of the development of resistance to low temperatures in the European corn borer (Kojić et al., 2010, 2018). In this regard, the increased concentration of UMP and NADP^+^ in the initial months of *O. nubilalis* diapause probably correlates with the processes of glycogen degradation, as well as the PPP and synthesis of glycerol, trehalose, and ATP. Our findings strengthen the established connection between the PPP pathway, glycogen metabolism and ADS in *O. nubilalis* diapause and cold hardiness development.

As respiration in individuals diapausing at low temperatures is known to be far lower than in non-diapausing ones (Wipking et al., 1995), low temperatures are likely to reduce the expression of examined genes for respiratory chain complexes, ATP synthesis and mitochondrial biogenesis, as evidenced in our study. Namely, the expression pattern of all analyzed energy-related genes – NADH dehydrogenase (*nd1*), CoQ-cytochrome *c* reductase (*uqcr*), *cox*, ATP synthase (*atp*), ADP/ATP translocase (*ant*), and prohibitin 2 (*phb2*), was the lowest in the first few months of diapause (*dca2* group) and then gradually increased during the time course of diapause. In the larval diapause of the cotton bollworm *H. armigera*, *cox* expression was lower compared to the non-diapausing group (Yang et al., 2010), and in the fat body of the firebug *Pyrrhocoris apterus*, a gradual decrease in the number of *cox* transcripts was determined during 120 days of reproductive diapause (Koštál et al., 2008). Expression of the *cox* gene does not change during the *E. scudderiana* diapause when compared to the non-diapausing group, although a trend of lower expression during mid-diapause can be observed (McMullen and Storey, 2008). However, in the larval diapause of the moth *O. fuscidentalis*, treatment with juvenile hormone analog has been shown to lead to increased oxygen consumption, COX enzyme activity and *cox1* gene expression, altogether resulting in the termination of diapause (Singtripop et al., 2007). Since prohibitin participates in mitochondrial biogenesis and proper respiratory chain formation, suppression of *phb2* expression at the onset of diapause at low temperatures may be due to active mitochondrial depletion in *O. nubilalis* larvae, a common mechanism of adaptations to low temperatures in insects (McMullen and Storey, 2008). Also, suppression may reflect adaptations to dormancy and prolongation of life during starvation in the *C. elegans* diapause (Artal-Sanz and Tavernarakis, 2009). Namely, the authors believe that prohibitin promotes longevity by modulating the use of lipids and energy production through the mitochondrial respiratory chain, as they proved this by using RNA interference to suppress the expression of p*hb1* and *phb2* in wild-type and mutants of the roundworm *C. elegans*. Reducing the expression of p*hb1/2* shortens the life of wild-type animals, but at the same time prolongs the life of individuals that are on caloric restriction, as well as mutants that cannot enter the so-called diapause or Dauer condition (Artal-Sanz and Tavernarakis, 2009). These findings on *C. elegans* are consistent with the results obtained in the present study, which showed that *phb2* expression was reduced at the onset of diapause at lower temperatures, probably as a form of adaptation to the ongoing starvation and the subsequent metabolic depression. Also, in most organisms, it is known that metabolic depression is the most pronounced in the first part of diapause, while the metabolism recovers over time, ahead of diapause termination. For this reason, during the latter part of diapause, an organism prepares to terminate this hypometabolic state by intensifying the process of mitochondrial biogenesis, in which prohibitin plays an important role (Coates et al., 1997). Metabolic recovery is further evidenced by the increase in the expression of genes whose products are involved in the regulation of energy metabolism, as evidenced in our study.

### Warm-Acclimation Maintains High Energy Expenditure and Leads to Premature Diapause Termination

This study has shown that energy metabolism in warm-acclimated diapausing larvae (*dwa2*) is distinct from cold acclimated diapausing groups due to higher COX activity, lower concentrations of NAD^+^/NADP^+^coenzymes, faster depletion of adenine nucleotides, and high expression of energy-related mitochondrial genes in the *dwa2* group. Moreover, UMP and GMP levels in these larvae are similar to those in the cold-acclimated *dca6* group whose metabolism is slowly recovering at low temperatures ahead of diapause termination. This is further supported by the PCA, which has clustered the *dwa2* group with the *dd* and *dca6* groups, with higher level of metabolic activity in comparison to *dca2* and *dca4*. Moreover, a similar level of energy metabolism-related gene expression between the *dwa2* and *dd* groups is most likely due to the development of larvae at high temperature (22 ± 1°C). In such conditions energy demands and metabolic rate remain at a high level, regardless of the fact that diapause-destined larvae are supposed to enter diapause due to a shorter photoperiod.

Given that warm-acclimated larvae (*dwa2* and *dd*) had increased gene expression for ADP/ATP translocase, high COX activity, and decreased ATP concentrations, we can assume that larval metabolism is kept at a high level, despite the fact that these larvae are either in preparation for dormancy (*dd*) or are already dormant (*dwa2*). As the European corn borer larvae do not feed during diapause, ATP is synthesized in smaller quantities and intensively consumed in comparison to active development (*nd*). Such conditions lead to depletion of ATP in the cytoplasm, which is probably compensated by increased expression of the ADP/ATP transporter gene. In addition, this gene also has a pattern of increasing expression during the time course of diapause, which indicates its importance for the metabolic recovery prior to diapause termination and resumption of active development. The obtained results of relative gene expression indicate that transcription is in line with the energy needs of *O. nubilalis* larvae in both warm- and cold-acclimated experimental groups.

### General Remarks

While at first glance diapause may seem like just a passive state of developmental arrest, the results of this and previous research on the European corn borer and other diapausing insects show that diapause is characterized by the maintenance of a dynamic equilibrium of energy metabolism. Furthermore, this balancing act is well synchronized with exogenous factors, such as the onset of low temperatures. This allows an organism to model its metabolism to respond to changes in the environment and direct it toward protective activities, such as synthesis of beneficial compounds (e.g., glycerol, trehalose, alanine, etc.) and removal of potentially harmful metabolites. Also, a gradual increase in the number of transcripts of all genes, and possibly their accumulation during diapause in cold conditions, may prepare the organism for termination of diapause and subsequent metamorphosis. After coming out of a long period of rest, during which metabolism was slowed down, mitochondrial biogenesis and reactivation of oxidative metabolism are necessary for rapid continuation of development, and numerous gene transcripts related to mitochondrial energy metabolism serve as a good source for protein synthesis.

The findings presented in this study evidence that diapause at high temperatures is shortened, stressful and leads to high mortality, while the exposure to low temperatures during diapause is substantial for long-term winter survival. Our results point to the possible adverse effects of the looming climate changes and global warming on species that are not accustomed to atypical temperature changes during specific stages of their life cycle, such as winter diapause.

## Dedication

We dedicate this article to our late professor Gordana Grubor-Lajšić (1949–2015), for her inspirational, passionate and dedicated work in the field of insect biochemistry and cold hardiness.

## Data Availability Statement

The raw data supporting the conclusions of this article will be made available by the authors, without undue reservation.

## Author Contributions

ŽP and VK designed the study. SG-D provided insect cultures. IU, MA, and ŽP performed gene expression analyses. VM and ŽP performed CE analysis. DB performed statistical analyses. ŽP, MA, and IU wrote the first draft of the manuscript. All authors contributed substantially to revisions.

## Conflict of Interest

The authors declare that the research was conducted in the absence of any commercial or financial relationships that could be construed as a potential conflict of interest.

## Publisher’s Note

All claims expressed in this article are solely those of the authors and do not necessarily represent those of their affiliated organizations, or those of the publisher, the editors and the reviewers. Any product that may be evaluated in this article, or claim that may be made by its manufacturer, is not guaranteed or endorsed by the publisher.
